# Siderophore-producing bacteria from Spitsbergen soils—novel agents assisted in bioremediation of the metal-polluted soils

**DOI:** 10.1007/s11356-024-33356-0

**Published:** 2024-04-23

**Authors:** Małgorzata Majewska, Anna Słomka, Agnieszka Hanaka

**Affiliations:** 1 Department of Industrial and Environmental Microbiology, Institute of Biological Sciences, Faculty of Biology and Biotechnology, Maria Curie-Skłodowska University, Akademicka 19, 20-031 Lublin, Poland; 2 Department of Plant Physiology and Biophysics, Institute of Biological Sciences, Faculty of Biology and Biotechnology, Maria Curie-Skłodowska University, Akademicka 19, 20-031 Lublin, Poland

**Keywords:** Siderophores, Iron-chelating molecules, Bioleaching agents, CAS-agar plate assay, Metal chelation efficiency, Heavy metals

## Abstract

Siderophores are molecules that exhibit a high specificity for iron (Fe), and their synthesis is induced by a deficiency of bioavailable Fe. Complexes of Fe-siderophore are formed extracellularly and diffuse through porins across membranes into bacterial cells. Siderophores can bind heavy metals facilitating their influx into cells via the same mechanism. The aim of the studies was to determine the ability of siderophore-producing bacteria isolated from soils in the north-west part of Wedel Jarlsberg Land (Spitsbergen) to chelate non-Fe metals (Al, Cd, Co, Cu, Hg, Mn, Sn, and Zn). Specially modified blue agar plates were used, where Fe was substituted by Al, Cd, Co, Cu, Hg, Mn, Sn, or Zn in metal-chrome azurol S (CAS) complex, which retained the blue color. It has been proven that 31 out of 33 strains were capable of producing siderophores that bind to Fe, as well as other metals. Siderophores from *Pantoea* sp. 24 bound only Fe and Zn, and *O. anthropi* 55 did not produce any siderophores in pure culture. The average efficiency of Cd, Co, Cu, Mn, Sn, and Zn chelation was either comparable or higher than that of Fe, while Al and Hg showed significantly lower efficiency. Siderophores produced by *S. maltophilia* 54, *P. luteola* 27, *P. luteola* 46, and *P. putida* 49 exhibited the highest non-Fe metal chelation activity. It can be concluded that the siderophores of these bacteria may constitute an integral part of the metal bioleaching preparation, and this fact will be the subject of further research.

**Graphical Abstract:**

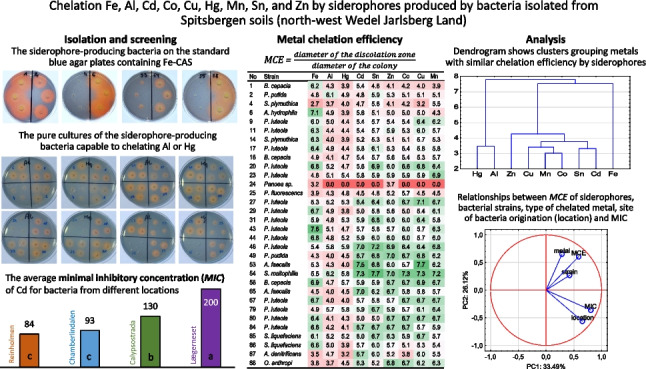

**Supplementary Information:**

The online version contains supplementary material available at 10.1007/s11356-024-33356-0.

## Introduction

Microorganisms produce various molecules that bind heavy metals (HM), such as amino acids (e.g., methionine and cysteine), peptides, proteins, low molecular weight organic acids (e.g., citric acid, tartaric acid, and oxalic acid), phenolic compounds, surfactants, bacterial pigments, polymeric extracellular substances, and metallophores (Budzikiewicz 2010; Kumar and Saxena 2020). Metallophores are classified into different groups based on their affinity for specific metals. For example, siderophore binds to Fe, chalcophore to Cu, manganesophore to Mn, nickelophore to Ni, and zincophore to Zn (Smethurst and Shcherbik 2021; Mohsen et al. 2023). These chelators play a crucial role in the HM transformation pathways. The HM mobilization occurring in the natural environment or during controlled bioremediation treatments is the result of metal stabilization and mobilization processes, which are controlled by the interactions of physical, chemical, and biological phenomena.

Under iron-deficient environment, many microorganisms produce siderophore, i.e., small molecular weight ligands (typically 1 kDa). These iron-chelating molecules are reported to increase Fe bioavailability and its transport across microbial cell membranes (Patel et al. 2018; Passari et al. 2023). In addition, it has been demonstrated that siderophore production may be induced by the presence of HM at high concentrations, and the binding of other metals, e.g., Al, Ag, Cd, Co, Cr, Cu, Hg, Ni, Mn, Pb, and Zn (Schalk et al. 2011; Saha et al. 2016; Patel et al. 2018), has been extensively studied. For example, the catecholate siderophores produced by *Burkholderia* sp. SX9 displayed a strong ability to metal mobilization and bonding Fe, as well as Zn and Cd (Wang et al. 2022a). Extracellularly formed HM-siderophore complexes may behave in two ways: (1) their diffusion through porins can be stopped causing them to remain outside the bacterial cells, or (2) receptors with low specificity may fail to differentiate between HM-siderophore and Fe-siderophore complexes, allowing for the uptake and accumulation of HM inside the cells (Schalk et al. 2011). Hesse et al. (2018) found a significant increase in the production and secretion of siderophores in HM-contaminated soils compared to uncontaminated soil. These findings suggest that microbial tolerance of toxic metals may involve many strategies, with siderophore production and secretion being one of them (Schalk et al. 2011).

Plants and their associated microorganisms (prokaryotes and microeukaryotes) exchange nutrients with each other. The microorganisms utilize organic compounds secreted by roots, while plants uptake the mineral nutrients mobilized by rhizosphere microbiota (Korenblum et al. 2022). Although plants can produce their own Fe-chelating substances (phytosiderophores), they effectively utilize microbial Fe-siderophore complexes (Kumar and Saxena 2020; Hanikenne et al. 2021). Złoch et al. (2016) revealed that approximately 47% of the rhizosphere bacteria were able to produce siderophores, which improved the Fe uptake by *Betula pendula* and *Alnus glutinosa* growing in the HM-contaminated soil. When plants are exposed to high HM concentrations, microbial siderophores can increase availability not only for the biologically essential metals but also for toxic ones, leading to their bioaccumulation. This phenomenon is used in phytoremediation, whereby plants are applied to clean contaminated sites. Microbe-assisted phytoremediation can eliminate toxic metals without negative effects on the soil structure (phytoextraction) or render them harmless (phytostabilization). The sustainable environmental remediation technologies are expected and widely accepted (Khan et al. 2020; Kumar and Saxena 2020; Pecoraro et al. 2022).

The significance of siderophores and siderophore-producing microorganisms (SPMs) in the biotechnological applications is closely related to their environmental roles. In both clean and polluted environments, SPMs control biogeochemical cycling of Fe and other metals through soil mineral weathering and mobilization and to make them more available to microorganisms and plants (Ahmed and Holmström 2014; Mosa et al. 2016; Pecoraro et al. 2022). Furthermore, SPMs can effectively counteract pathogens and help plants in mitigating HM stress by decreasing the concentration of reactive oxygen species (Ahmed and Holmström 2014; Saha et al. 2016; Pecoraro et al. 2022). Additionally, siderophores are known as bioleaching agents. As evidenced by Williamson et al. (2021), siderophores possess the potential to bioleach Zn, Mn, and Al from low-grade primary ores and secondary mineral residues. Their research represents a significant advancement toward the development of sustainable metal extraction systems and biometallurgical technologies. Siderophores also have important applications in medicine as the antimicrobial agents (antibiotics), in Fe overload therapy and against *Plasmodium falciparum* (antimalarial activity) (Saha et al. 2016). Siderophores can be used for targeted drug delivery (antibiotic and anticancer therapy), because the siderophore-drug conjugate is able to transfer more easily across the cell membrane by binding to siderophore receptors (Pecoraro et al. 2022).

Several assays have been developed to detect SPMs. Quantitative methods that determine the amounts of total Fe-chelators as well as catechol and hydroxamate siderophores in the growth medium of microorganisms are employed. For example, reactions with FeCl_3_ are useful in estimating all possible Fe-chelators (Atkin et al. 1970). Moreover, the amounts of catechol or hydroxamate siderophores can be determined by the Arnow method with 3,4-dihydroxybenzoic acid as the standard (Arnow 1937) or the Csaky method with hydroxylamine hydrochloride as the standard (Csaky 1948), respectively. In order to visually identify SPMs, the blue agar containing chrome azurol S (CAS) may be used. The pink-orange zones around the colonies after incubation are recognized as a positive effect, namely, the production of siderophores (Schwyn and Neilands 1987).

Due to their considerable potential for bioremediation, siderophores are the subject of this research paper. The bacteria isolated from the Spitsbergen soils (Hanaka et al. 2019a, b) were studied to determine their capability to produce siderophores to chelate metals other than Fe (non-Fe metals). The most efficient siderophore-producing isolates (33 strains) were cultivated on the modified blue agar plates, where Fe in CAS solution was substituted with other metals (Al, Cd, Co, Cu, Hg, Mn, Sn, and Zn).

## Materials and methods

### Soil origin and isolation of bacteria

The soil samples originated from four areas: Calypsostranda (CAL), Chamberlindalen (CH), Lægerneset (LN), and Reinholmen (REIN) located in the north-west part of Wedel Jarlsberg Land on the southwestern Spitsbergen (Hanaka et al. 2019a, b). These incipient soils with poorly formed profiles were generally unpolluted with heavy metals. However, the geoaccumulation index (*I*_geo_) of Cd ranged from 1.05 to 1.43 and the Cd enrichment factor (EF) ranged from 2.20 to 2.91 which were found in the CAL, LN, and REIN areas and were significantly higher than in the CH soils (Hanaka et al. 2019b). Taking into account the values of *I*_geo_ > 1 and EF > 2, these soils were considered to be moderately contaminated with Cd (Barbieri 2016).

The siderophore-producing microorganisms were isolated from these soils using the standard blue agar with chrome azurol sulphonate (CAS) as described by Schwyn and Neilands (1987). After the soil suspensions were subjected to a tenfold dilution series, 0.1 ml of the appropriate dilution was pipetted onto the blue agar surface and spread evenly with a sterile glass rod. After incubation at 20 °C for 7 days, the bacteria showing a pink-orange zone around their colonies were recognized as producers of siderophores. Of the total, 33 isolates (Table 1) with the largest zones of discoloration were selected for further testing. These isolates were purified and stored in 50% glycerol solution at − 80 °C until analysis. Their biochemical characterization was conducted using the API20NE test in accordance with the manufacturer’s instruction (bioMérieux Inc., Marcy-l’Etoile, France). After 24 and 48 h of analysis, the database (apiweb.biomerieux.com) was used to indicate similarity to reference strains (Hanaka et al. 2019a).

**Table 1:** Siderophore-producing bacteria: biochemical level of similarity (%ID) and similarity index (*T*) according to API20NE test, the sampling sites (Hanaka et al. 2019a, b), and the minimal inhibitory concentration (MIC) of Cd (µg ml^−1^)

Sampling site	Symbol	Bacteria	%ID	*T*	MIC
Calypsostranda	Sp14	*Serratia plymuthica*	99.9	0.48	80
Calypsostranda	Pl17	*Pseudomonas luteola*	92.5	0.56	100
Calypsostranda	Bc18	*Burkholderia cepacia*	80.1	0.60	80
Calypsostranda	Pl20	*Pseudomonas luteola*	92.5	0.56	200
Calypsostranda	Pl23	*Pseudomonas luteola*	92.5	0.56	200
Calypsostranda	P24	*Pantoea* sp.	82.3	0.22	20
Calypsostranda	Pf25	*Pseudomonas fluorescens*	99.9	0.97	40
Calypsostranda	Pl27	*Pseudomonas luteola*	92.5	0.56	200
Calypsostranda	Pl29	*Pseudomonas luteola*	99.8	0.62	80
Calypsostranda	Pl31	*Pseudomonas luteola*	98.8	0.62	100
Calypsostranda	Sl85	*Serratia liquefaciens*	99.8	0.38	200
Calypsostranda	Sl86	*Serratia liquefaciens*	95.9	0.43	200
Calypsostranda	Ad87	*Achromobacter denitrificans*	82.2	1.00	60
Calypsostranda	Oa88	*Ochrobactrum anthropi*	99.5	0.77	150
Chamberlindalen	Sp4	*Serratia plymuthica*	99.9	0.64	40
Chamberlindalen	Ah6	*Aeromonas hydrophila*	99.7	0.26	80
Chamberlindalen	Pl9	*Pseudomonas luteola*	99.8	0.62	150
Chamberlindalen	Pl11	*Pseudomonas luteola*	99.7	0.59	150
Chamberlindalen	Af65	*Alcaligenes faecalis*	90.1	0.91	60
Chamberlindalen	Pl67	*Pseudomonas luteola*	99.8	0.62	80
Lægerneset	Pl43	*Pseudomonas luteola*	99.8	0.62	200
Lægerneset	Pl44	*Pseudomonas luteola*	99.5	0.66	200
Lægerneset	Pl46	*Pseudomonas luteola*	99.8	0.62	200
Lægerneset	Pp49	*Pseudomonas putida*	99.1	0.63	200
Reinholmen	Bc1	*Burkholderia cepacia*	99.5	0.66	60
Reinholmen	Pp2	*Pseudomonas putida*	87.9	0.33	80
Reinholmen	Af53	*Alcaligenes faecalis*	90.1	0.91	20
Reinholmen	Sm54	*Stenotrophomonas maltophilia*	99.9	0.96	5
Reinholmen	Oa55	*Ochrobactrum anthropi*	96.5	0.63	20
Reinholmen	Bc58	*Burkholderia cepacia*	99.5	0.66	200
Reinholmen	Pl79	*Pseudomonas luteola*	92.5	0.56	100
Reinholmen	Pl80	*Pseudomonas luteola*	92.5	0.56	60
Reinholmen	Pl84	*Pseudomonas luteola*	99.8	0.62	150

Identification level: perfect %ID ≥ 99.9, *T* ≥ 0.75; very good %ID ≥ 99.0, *T* ≥ 0.50; good %ID ≥ 90.0, *T* ≥ 0.25; acceptable %ID ≥ 80.0, *T* ≥ 0.00

### MIC determination

Taking into account the Cd present in the tested soils (Hanaka et al. 2019b), the minimal inhibitory concentration (MIC) of Cd was determined. MIC was estimated from the visible growth of the bacteria at 20 °C. The bacterial suspensions (0.5 McFarland) in 0.9% sterile NaCl solution were inoculated to a liquid broth medium on the 96-well microplates in the presence of different Cd concentrations (from 5 to 200 µg ∙ ml^−1^). After 24 h of aerobic incubation, growth was assessed.

### Preparation of blue agar plates

The preparation process for the blue agar plates requires various solutions (Schwyn and Neilands 1987): (1) PIPES solution, which involves dissolving 6 g of NaOH in 1000 ml of H_2_O and slowly adding 32.24 g of piperazine-N,N′-bis(2-ethanesulfonic acid) while maintaining the pH value up to 6.8; (2) minimal medium, which includes dissolving 3.0 g of KH_2_PO_4_, 0.5 g of NaCl, 1.0 g of NH_4_Cl, 0.02 g of MgSO_4 _∙ 7H_2_O, and 4.0 g of glucose in 860 ml of PIPES. Add 15.0 g of agar and sterilize by autoclaving (121 °C, 760 hPa, 20 min); then, prepare (3) CAS solution by dissolving 0.06 g of CAS (C_23_H_13_Cl_2_Na_3_O_9_S) in 50 ml of H_2_O, adding 10 ml of 1 mM FeCl_3 _∙ 6H_2_O in 10 mM HCl, along with 40 ml water solution of hexadecyltrimethylammonium bromide (HDTMA). After sterilization by autoclaving (121 °C, 760 hPa, 20 min) and cooling down to 50 °C, 30 ml of 10% casamino acid, 10 ml of 0.01 M CaCl_2_, and CAS solution were added to the minimal medium. The final solution was thoroughly mixed and the plates were aseptically poured.

To screen siderophores with chelating properties for non-Fe metals, several CAS solutions were prepared by replacing FeCl_3 _∙ 6H_2_O with chloride salts of the other metals (Al, Cd, Co, Cu, Hg, Mn, Sn, Zn). The concentration of all metal ions was maintained at a constant level of 1 mM metal solution in 10 mM HCl, and the instant color change of the prepared solution was similar to that of the conventional CAS solution (Patel et al. 2018). Each of these modified CAS solutions was used to prepare blue agar plates. It was noticed that in order to obtain a blue color of the modified blue agar containing Cd, Co, Cu, Mn, Sn, and Zn, it was necessary to increase the pH of the medium from 6.8 to 7.1. Under these conditions, the modified agar achieved a similar color to that of standard blue agar.

### Determination of binding of siderophores to non-Fe metals

To prepare the cell suspension for inoculation, the bacteria were cultivated in a broth medium (standard liquid medium used for the cultivation of a wide variety of microorganism) at 20 °C for 24 h. Then, the bacterial cultures were centrifuged (10,000 rpm, 20 min, 4 °C), and the resultant cell pellets were washed twice with sterile distilled water before resuspension in 10 ml of 0.9% sterile saline solution. The number of cells in the inoculum was determined using the McFarland scale (OD550) and diluted to 1,000,000 cells in 1 ml. Next, 5 µl of the appropriate inoculum was spotted on the blue agar plates, which contained modified CAS solution for each metal. The appearance of a pink-orange halo around the colonies after 7 days at 20 °C, much like in the original method, was identified as a positive effect, indicating the production of siderophores on the modified blue agar. The metal chelation efficiency (MCE) of the siderophores excreted by each strain was calculated as a ratio of the diameter of the discoloration zone of the blue medium to the diameter of the colony.

### Statistical analysis

The data were subjected to statistical analysis using Statistica 13.3 software. Analysis of variance (one-way ANOVA) with a post hoc Tukey’s test at *p* < 0.05 was performed. To demonstrate the similarity of chelation efficiency (CE) among the tested metals, the cluster analysis was carried out. Additionally, the principal component analysis (PCA) was undertaken to show the relationship between MCE of siderophores, bacterial strains, the type of chelated metal, the place of origin of the strains, and MIC. The mean value of MCE secreting siderophores by each strain was presented (*n* = 3) and visualized as a heat map (Microsoft Excel 2016).

## Results

**Fig. 1:**
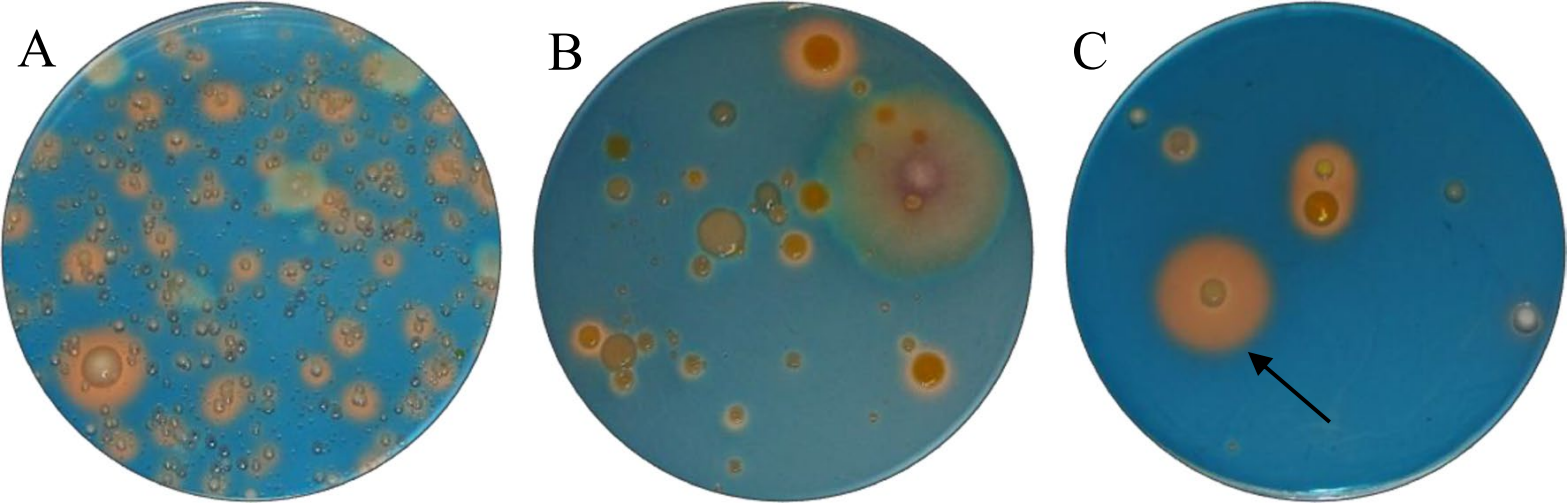
Colonies of the soil microorganisms growing on the standard blue agar with chrome azurol sulphonate (CAS) and the pink-orange zones indicating the production of siderophores. Agar media were inoculated with the diluted soil suspension: **A** 1000-fold dilution; **B** 10,000-fold dilution; **C** 100,000-fold dilution. The arrow indicates the colony with the highest siderophore diffusion zone

A total of 33 bacterial isolates were obtained from the Spitsbergen soils by a tenfold dilution series and incubation on standard blue agar (Fig. 1). Next, they were purified on nutrient agar plates, and an analysis of their metabolic profiles was performed using the API20NE test. The analysis revealed biochemical similarity of the isolates to 12 distinct species, representing 9 genera (Table 1). Fifteen of the isolates showed similarity to *Pseudomonas luteola*. The tested strains showed different Cd tolerance (Table 1). *Pseudomonas luteola* (strains 43, 44, 46) and *P. putida* (strain 49), which were isolated from LN soil, exhibited very high MIC levels (200 µg ml^−1^). Meanwhile, there was a greater difference in Cd tolerance among the isolates originating from CAL and CH (ranging from 20 to 200 µg ml^−1^), as well as those from REIN (ranging from 5 to 200 µg ml^−1^).

**Fig. 2:**
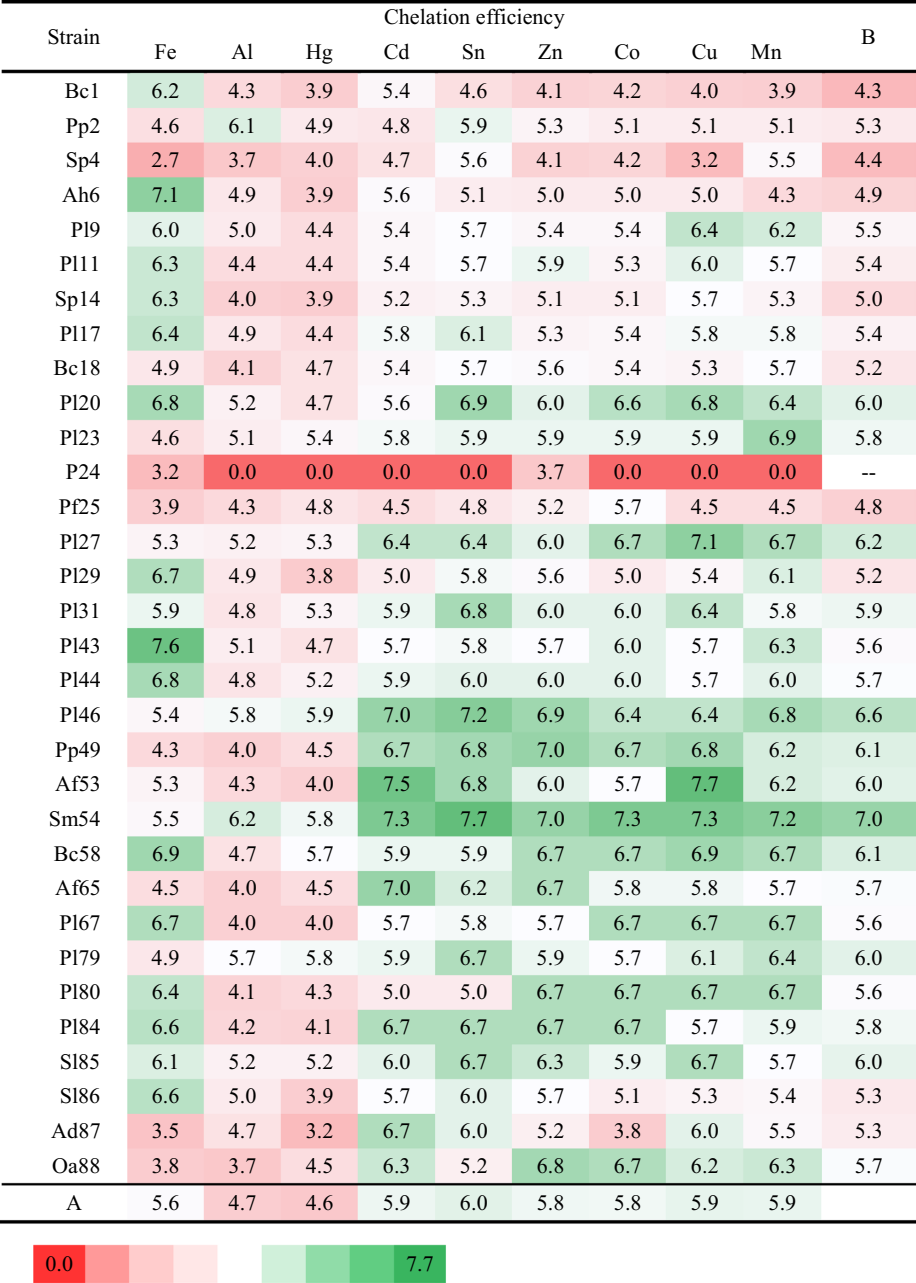
Heat map representing the metal chelation efficiency (MCE) of siderophores produced by bacteria isolated from Spitsbergen soils. **A** The average chelation efficiency calculated for individual metals. **B** The average non-Fe metal chelation efficiency calculated for individual strains

All isolates were screened for siderophore production using standard blue agar containing Fe-CAS complex. The tested strains formed colonies with varying diameters (ranging from 2 to 5 mm), and their siderophores diffused in the agar at differing distances from the colony (ranging from 2 to 25 mm). In most cases, the created haloes were either orange or pink, although a very narrow, bright yellow halo was found around the colony of *Pantoea* sp. 24. The Fe and non-Fe CE of siderophores was calculated by measuring the size of the colony and the resulting halo formed around it. Significant differences in the ability to synthesize siderophores were observed among selected strains according to the CAS agar diffusion assay (Fig. 2). The highest Fe-CE was revealed for *P. luteola* 43 (7.6) and *A. hydrophila* 6 (7.1). Lower, but also significantly high, Fe-CE (6.7–6.9) were determined for the siderophores produced by *B. cepacia* 58 and strains 20, 29, 44, 67 of *P. luteola*. By contrast, the lowest Fe-CE were noted for *S. plymuthica* 4 (2.7) and *Pantoea* sp. 24 (3.2), while no visible halo was observed around colonies of *O. anthropi* 55.

To screen for bacteria that produce siderophores capable of chelating non-Fe metals, plates with modified blue agar were prepared. Complex Fe-CAS was replaced individually with Al-CAS, Cd-CAS, Co-CAS, Cu-CAS, Hg-CAS, Mn-CAS, Sn-CAS, or Zn-CAS. After 7 days of growth, tested strains were observed to have turned the modified CAS-agar from blue to pink/orange (Supplementary Information, Fig. S1). This fact indicated the production of siderophores and chelation of non-Fe metals. However, the alteration in the diameter of the discoloration zone was a unique trait of each strain and was linked to the type of metal added to the medium. *Pantoea* sp. 24 stopped the synthesis of siderophores in the presence of Cd-CAS, Co-CAS, Cu-CAS, Mn-CAS, and Sn-CAS, except for Zn-CAS. The Al-CE and Hg-CE of siderophores produced by 19 strains were significantly lower than of Fe; however, in the case of 8 strains, it was comparable to Fe, while for 4 strains it was higher (*P. putida* 2, *S. plymuthica* 4, *P. luteola* 79, *A. denitrificans* 87). Furthermore, Cd-CE, Co-CE, Cu-CE, Mn-CE, Sn-CE, and Zn-CE of siderophores produced by *B. cepacia* 1, *A. hydrophila* 6, *S. plymuthica* 14, *P. luteola* 29, *P. luteola* 43, *P. luteola* 44, and *S. liquefaciens* 86 were significantly decreased when compared to Fe-CE. On the other hand, significantly higher CE of Cd, Co, Cu, Mn, Sn, and Zn compared to Fe was determined for siderophores produced by *S. plymuthica* 4, *B. cepacia* 18, *P. luteola* (strains 23, 27, 46, 79), *P. putida* 49, *A. faecalis* 53 and 65, *S. maltophilia* 54, *A. denitrificans* 87, and *O. anthropi* 88 (SI, Fig. S2).

**Fig. 3:**
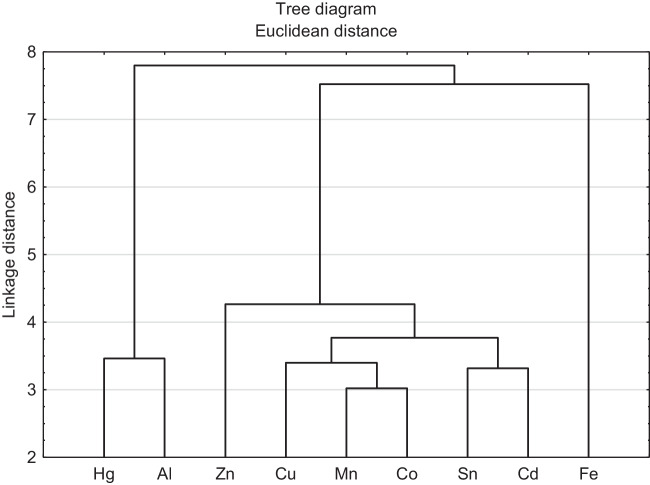
Tree diagram from cluster analysis for means of the chelation efficiency (CE) of Fe and non-Fe metals: Al, Cd, Co, Cu, Hg, Mn, Sn, and Zn

**Fig. 4:**
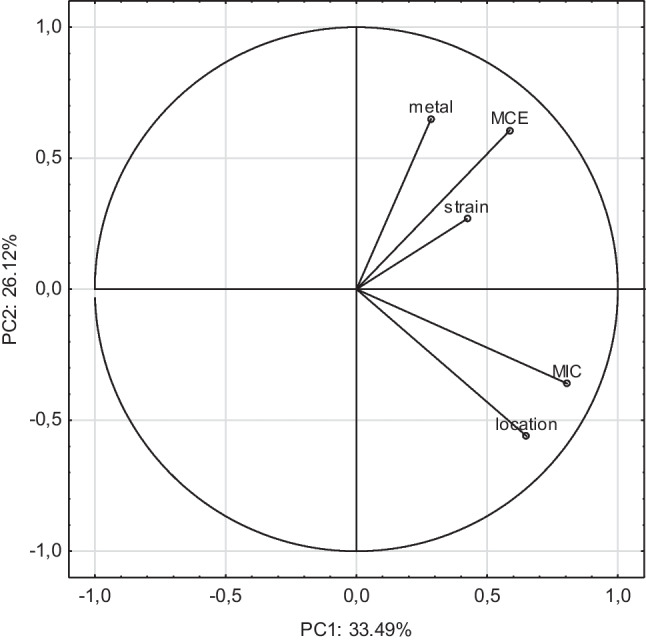
Diagram of the principal component analysis (PCA), describing the relationship between the metal chelation efficiency (MCE) of siderophores, the bacterial strains, type of chelated metal (Fe, Al, Hg, Co, Cd, Cu, Mn, Sn, and Zn), site of origination of bacteria (location), and minimal inhibitory concentration (MIC) of Cd

The cluster analysis allowed to determine which metals were chelated with similar efficiency by siderophores. It turned out that Fe was found in a separate cluster of dendrogram (Fig. 3). In the second cluster, Hg and Al were classified as having the most similar chelation efficiency, with the remaining metals (Co, Cd, Cu, Mn, Sn, and Zn) grouped into the third cluster. The first two principal components in PCA explained 59.61% of the total variations among the samples (Fig. 4). PC2 facilitated the separation of the chelated metal, MCE, MIC, the type of bacterial strain, and the strain location. The type of chelated metal, MCE, and strain type were positively while MIC and strain location negatively correlated with PC2. PCA showed that the MCE of siderophores was significantly influenced by the type of chelated metal and the type of bacterial strain (Fig. 4). In contrast, the MIC was determined by the bacteria location.

**Fig. 5:**
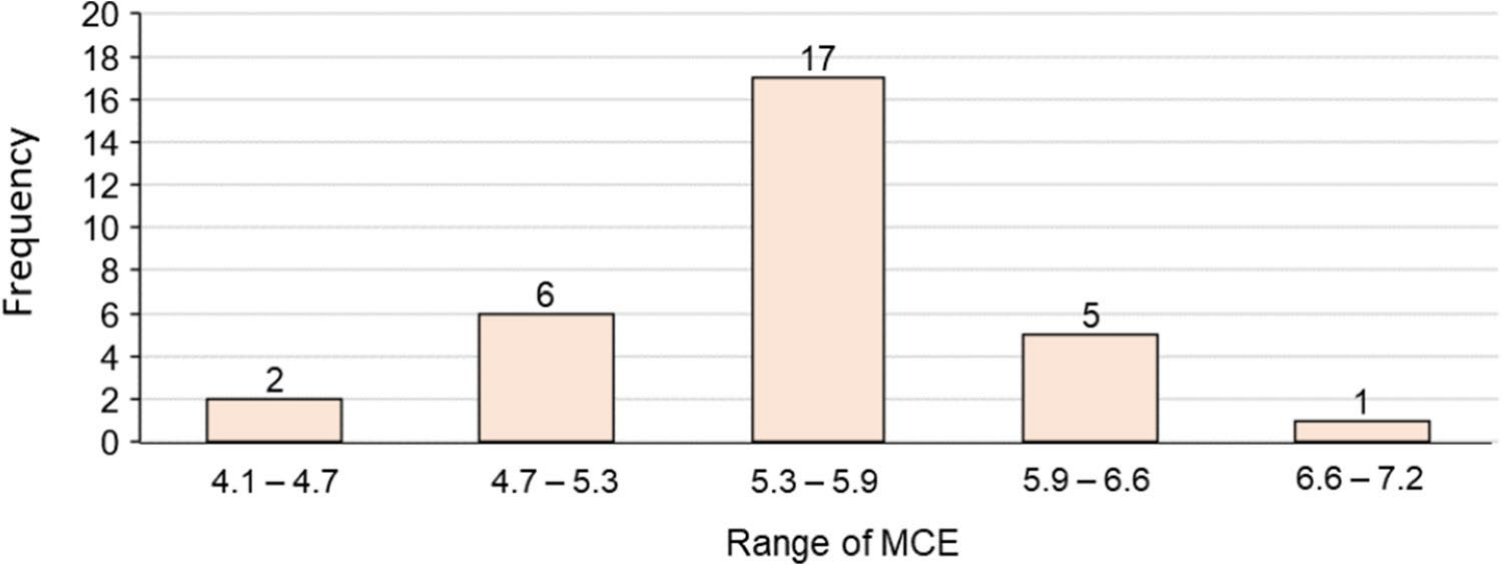
Frequency histogram describing distribution for the average values for the metal chelation efficiency (MCE) of siderophores produced by bacteria isolated from Spitsbergen soils

**Fig. 6:**
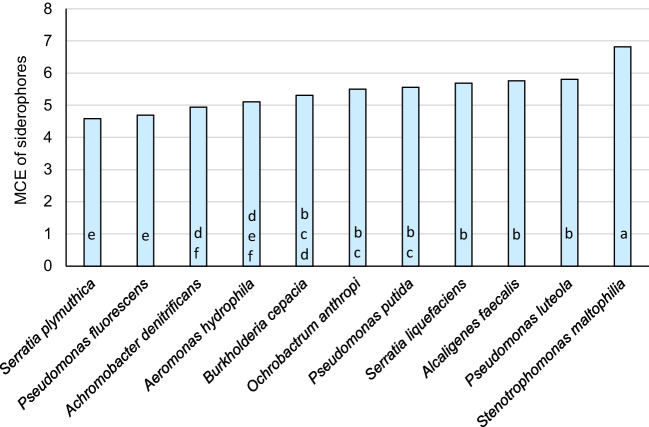
The average metal chelation efficiency (MCE) of siderophores produced by bacterial strains belonging to particular species

The siderophores synthesized by the tested strains differed in MCE. Of the total, 17 had MCE ranging from 5.3 to 5.9, 8 had MCE lower than 5.3, and 6 had MCE higher than 5.9 (Fig. 5). The higher average value of MCE was calculated for *S. maltophilia* 54 (6.8). Differences were evident among strains belonging to different genera, as well as within a genus (Fig. 6). For example, the siderophores produced by *P. luteola* and *P. putida* (5.8 and 5.6, respectively) were more efficient in chelating metals than *P. fluorescens* (4.7), and *S. liquefaciens* (5.7) was more efficient than *S. plymuthica* (4.6). It can be assumed that siderophores produced by *S. maltophilia*, *P. luteola*, A*. faecalis*, *S. liquefaciens*, and *P. putida* exhibited the highest activity in chelating non-Fe metals.

## Discussion

The research of Hanaka et al. (2019a, b) showed that Spitsbergen soils despite pioneering conditions are biologically active and are the source of microorganisms which could be useful for various purposes (e.g., promoting and regulating plant physiology by the production of siderophores, indole-3-acetic acid, and 1-aminocyclopropane-1-carboxylate deaminase). It was observed that 72% of the bacterial isolates from these soils synthesized siderophores, and an increased number of SPMs was significantly correlated with a lower amount of Fe in the soil (Hanaka et al. 2019a). Due to higher values of *I*_geo_ and EF for soil Cd, many of the tested isolates tolerated the highest Cd concentration in in vitro tests (MIC, 200 µg ml^−1^) and produced siderophores that exhibited the high chelation efficiency for non-Fe metals (Table 1, Figs. 5 and 6).

As previously demonstrated by Shin et al. (2001), the CAS-agar plate assay is a simple and highly reproducible method. It was recommended for screening and preliminary siderophore analysis, as only siderophores not bound to Fe decolorized the blue Fe-CAS complex. Subsequent studies proved the possibility of replacing Fe in the Fe-CAS complex with the non-Fe metal (e.g., Ni, Mn, Zn, Cu, Co, Hg, Ag) and evaluating the efficiency of non-Fe metal complexation (Patel et al. 2018). We noticed that the halos around the colonies of isolates tested on the blue agar vary in shades of pink or orange (SI, Fig. S1). The intensity of the color zone was characteristic of the strain regardless of the metal present in the medium. This color change reaction could be most likely related to the chemical structure or concentration of the secreted siderophores (Koedam et al. 1994; Milagres et al. 1999), as well as acidification of the medium (Shin et al. 2001; Hofmann et al. 2021). According to scientific studies, the color of the observed zones can range from reddish-purple (Milagres et al. 1999; Srivastava et al. 2018), magenta (Srivastava et al. 2018), orange (Milagres et al. 1999; Shin et al. 2001) to yellow and colorless (Patel et al. 2018; Singh et al. 2020; Hofmann et al. 2021). It has been suggested that these colors are indicative of specific class of siderophores. Blue agar is known to be discolored to pink by catechols and to orange by hydroxamates, while yellow halos (Hofmann et al. 2021) can indicate the production of both siderophores and acidifying non-typical chelators such as citric and oxalic acids (Srivastava et al. 2018). Observations of intermediate colors, between pink and orange, may be associated with the production of several types of siderophores by a strain. Some bacteria are capable of synthesizing a wide range of siderophores. For example, *P. fluorescens* produce siderophores including ferribactin, pyoverdine, pyochelin, pseudobactin, and ornicorrugatin (Budzikiewicz 2010; Schalk et al. 2011; Saha et al. 2016; Williamson et al. 2021), *B. cepacia*—cepabactin, pyochelin, ornibactin, cepaciachelin, and azurochelin (Budzikiewicz 2010; Schalk et al. 2011; Saha et al. 2016), *P. luteola* (synonym *Chryseomonas luteola*)—chrysobactin and chryseomonin (Adolphs et al. 1996; Budzikiewicz 2010). On the contrary, some strains produce a single dominant type of siderophore, e.g., *A. hydrophila*—amonabactin (Budzikiewicz 2010), *S. maltophilia* K279a—2,3-dihydroxybenzoylserine (Hisatomi et al. 2021), *Pantoea brenneri*—enterobactin-like siderophore (Suleimanova et al. 2023), and *S. plymuthica*—serratiochelin C (Restrepo et al. 2021).

Generally, siderophores are molecules with high specificity for Fe, but can also bind non-Fe metals (Mosa et al. 2016; Egamberdieva and Ahmad 2018; Khan et al. 2020; Wang et al. 2022a). The present study also demonstrated this phenomenon (Table 1, Figs. 2 and 6, SI Fig. S2). High concentrations of toxic metals in polluted environments can mask the presence of available Fe, leading to Fe deficiency in cells (Dimkpa et al. 2012; Hussein and Joo 2014; Mosa et al. 2016; Złoch et al. 2016; Zhang et al. 2023). Therefore, despite the abundance of Fe, bacteria may increase siderophore production by up to 100% (Egamberdieva and Ahmad 2018). In addition, Patel et al. (2018) proved that the siderophores produced by *Alcaligenes* sp. RZS2 and *P. aeruginosa* RZS3 could remove Ni or Mn from modified CAS agar plates with greater chelation strength than for Fe. The regulation of siderophore synthesis is based on the Fe content in the medium. As Fe-CAS releases Fe, an increase in Fe concentration can cause a decrease in the production of siderophores (Srivastava et al. 2018). The lowest MCE by siderophores was observed in media containing Al-CAS or Hg-CAS (Fig. 2). Bacterial cells probably captured the Al- and Hg-siderophore complexes, which had a toxic effect on the synthesis or secretion of siderophores. For instance, *Azotobacter vinelandii* regulates the production of azotochelin in the presence of Mo. Azotochelin production was activated up to 100 µM Mo in the medium, whereas > 100 µM Mo completely repressed the synthesis of this siderophore (Schalk et al. 2011). The formation of complexes with toxic metals can have dual aspects. Firstly, if the receptors have low specificity and cannot distinguish between HM complexes and Fe complexes, intracellular accumulation of HM may occur (Schalk et al. 2011; Khan et al. 2020). Secondly, the presence of HM-siderophore complexes in the extracellular medium can protect bacteria from these metals by sequestering them outside the cells (Schalk et al. 2011).

The estimated MCE varied depending on the isolate and the chelated metal. Generally, CE of Mn, Cu, and Sn was considerably higher, while Co, Zn, Cd, and Mn were comparable to Fe-CE. Only Hg-CE and Al-CE were lower than Fe and other metals. This phenomenon was observed for siderophores produced by *B. cepacia* 18, *P. luteola* (23, 27, 46, 49, 79), *S. maltophilia* 54, *A. faecalis* (53, 65), *A. denitrificans* 87, and *O. anthropi* 88. Therefore, these strains may be useful in bioaugmentation as a strategy for the bioremediation. Yu et al. (2017) used siderophore-producing *Bacillus* sp. PZ-1 to augment the phytoextraction of Pb from the soil. Introducing siderophores or SPMs into HM-contaminated soils can accelerate and enhance the effectiveness of natural detoxification processes. Hesse et al. (2018) found that adding Cu to the soil affected the structure of the bacterial community. The Cu presence resulted in the selection of the taxa at the genus level that produced large amounts of siderophore. As a result, the proportion of SPMs was significantly higher in Cu-contaminated soil compared to control soil. SPMs are a significant component of the microbial community in rhizosphere soils, ranging from 47 (Złoch et al. 2016) to 85% (Tian et al. 2009). In the rhizosphere, a mixture of microbial siderophores supports the Fe nutrition of plants, enhances the metal translocation from the soil to the plant (phytoextraction), and mitigates the stress induced by HMs and other toxins (Złoch et al. 2016; Kumar et al. 2017; Roskova et al. 2022).

Under appropriate conditions, siderophores can support the stabilization or extraction of various metals during bioremediation. The siderophores of *Nocardioides simplex* 3E, *Rhodococcus erythropolis* B7g, when immobilized on the C18 solid-phase columns, have demonstrated an efficient sorption of V, Ga, and In from mixed metal solutions (Hofmann et al. 2021). Furthermore, positively charged HM-siderophore complexes (e.g., Pb, Zn, and Cd) can be effectively sorbed onto negatively charged zeolite surface and immobilized as descripted Karimzadeh et al. (2013). Also, biosorption is a crucial factor in the neutralization of toxic metals. For example, Khan et al. (2020) observed that *Bacillus subtilis* intracellularly accumulated Cd when hydroxamate siderophores produced by *Aspergillus nidulans* were present. This cross-facilitation of metal bioaccumulation may be of particular importance in phytoextraction supported by exogenous siderophores. This is crucial since plant siderophores (phytosiderophores) usually have a lower (10–30 times) affinity for Fe (Zaidi et al. 2012) and are often present in lower concentrations in the rhizosphere compared to the microbial siderophores (Egamberdieva and Ahmad 2018). Although the HM-siderophore complexes may not be absorbed by the plant and remain in the aqueous phase temporarily (Ahmed and Holmström 2014), they can decrease the toxicity of the metal (Złoch et al. 2016), support seed germination (Wang et al. 2022a) or plant growth (Kumar et al. 2017; Wang et al. 2022b), and finally favor the revitalization of the plant cover.

Siderophores have a strong solubilizing effect capable of contributing to the weathering of rocks and minerals in the environment. They are also implicated in the leaching of HM from contaminated soils or ores. For example, Williamson et al. (2021) proved the potential of siderophores produced by *Pseudomonas* strains (*P. fluorescens*, *P. azotoformans*, and *P. putida*) to bioleach Zn, Mn, and Al from low-grade ores and secondary minerals. Moreover, siderophores of *Burkholderia* sp. SX9 were able to mobilize Cu, Zn, and Cd from insoluble oxides or carbonates and effectively formed soluble HM-siderophore complexes (Wang et al. 2022a). These studies are considered important for the development of sustainable metal extraction systems and biometallurgical technologies.

It is evident from our findings that siderophores and SPMs are crucial in bioremediation and phytoremediation. Therefore, further studies should be continued to optimize SPM culture conditions, improve the efficiency of siderophore production, and develop methods of isolation and purification for metal mobilizing compounds. These outcomes could be used to promote plant growth in contaminated areas, thus facilitating bioremediation and renewal of plant cover.

## Conclusions

Deficiency of Fe induces the synthesis of siderophores. In HM-contaminated environments as a result of insufficient iron resources, siderophores may be continually produced and used to also chelate non-Fe metals. The efficiency of chelating non-Fe metals, which are essential micronutrients (Co, Cu, Mn, Sn, Zn), was comparable or higher than that of Fe. However, toxic Al and Hg were chelated with significantly lower efficiency. The average MCE and MIC strains isolated from LN soil were much higher than strains isolated from other locations (CAL, CH, REIN). Siderophores produced by *S. maltophilia* 54, *P. luteola* 27, *P. luteola* 46, and *P. putida* 49 were the most active in chelating non-Fe metals (MCE > 6.0) than Fe. These strains will be the subject of further study including optimization of cultivation and siderophore production. Additionally, preparation containing bacterial siderophores will be developed and their potential use in HM bioleaching will be explored.

### Supplementary Information

Below is the link to the electronic supplementary material.

Supplementary file1 (DOCX 10503 KB)

## Data Availability

The datasets used and/or analyzed during the current study are available from the corresponding author on reasonable request.
