# Supplementary material for: Siderophore-producing bacteria from Spitsbergen soils—novel agents assisted in bioremediation of the metal-polluted soils
Source: Environ Sci Pollut Res Int. 2024 Apr 23;31(22):32371–81. doi: 10.1007/s11356-024-33356-0 (PMC11133149; doi:10.1007/s11356-024-33356-0)
Supplement: Supplementary file 1 — Supplementary file1 (DOCX 10503 KB) [file 11356_2024_33356_MOESM1_ESM.docx]

**Siderophore-producing bacteria from Spitsbergen soils – novel agents assisted in bioremediation of the metal-polluted soils**

Małgorzata Majewska^*1^, Anna Słomka^1^ Agnieszka Hanaka^2^

^1^ Department of Industrial and Environmental Microbiology, Institute of Biological Sciences, Faculty of Biology and Biotechnology, Maria Curie-Skłodowska University, Akademicka 19, 20-031 Lublin, Poland;

^2^ Department of Plant Physiology and Biophysics, Institute of Biological Sciences, Faculty of Biology and Biotechnology, Maria Curie-Skłodowska University, Akademicka 19, 20-031 Lublin, Poland

*Correspondence: malgorzata.majewska@mail.umcs.pl

**SUPPLEMENTARY INFORMATION**


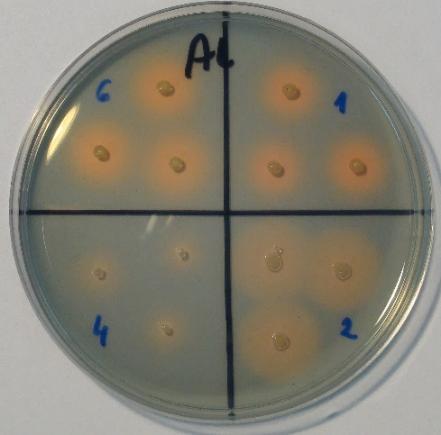

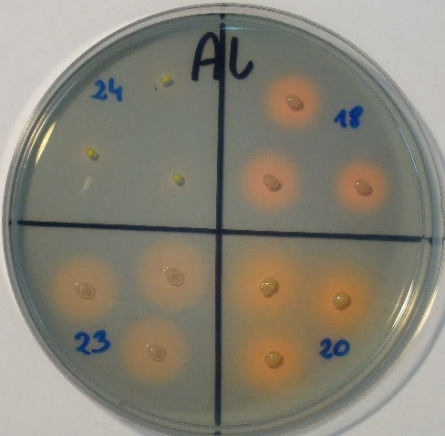

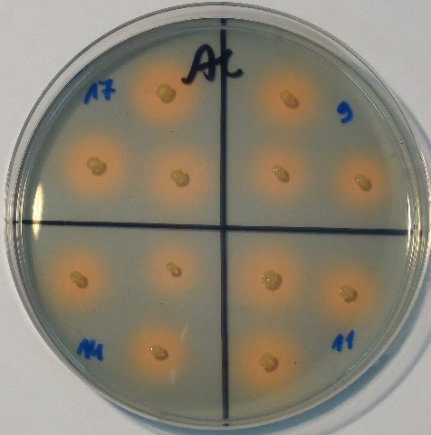

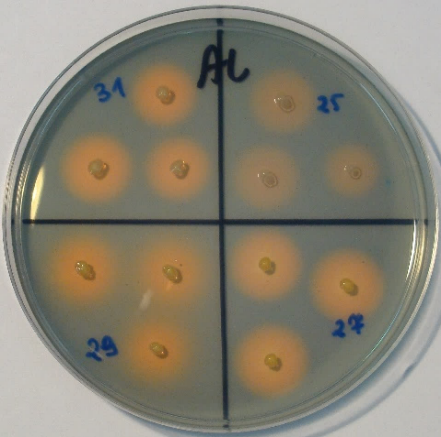

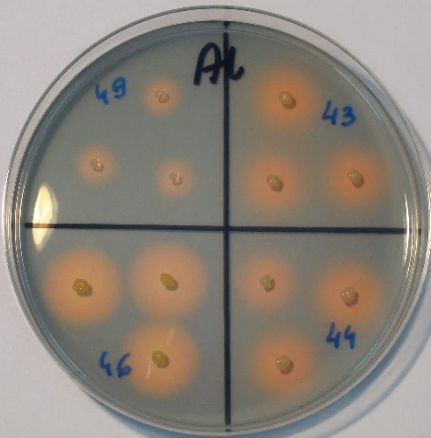

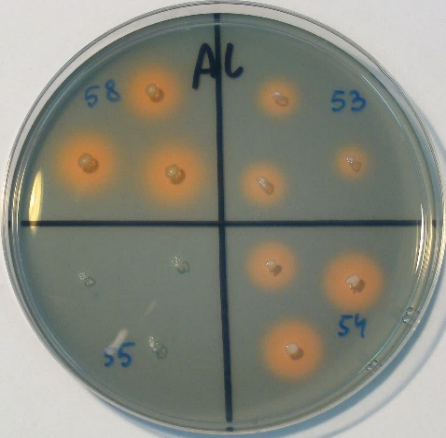

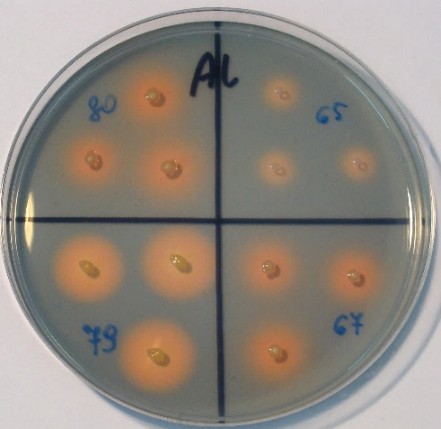

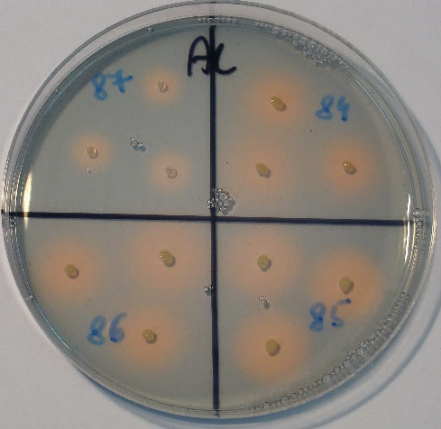


**Fig. S1** Production of siderophores by soil bacteria growing on the modified blue agar plates, where iron (Fe) was substituted by aluminum (Al)

**Fig. S2** The metal chelation efficiency (MCE) of siderophores produced by bacteria from Spitsbergen soils

c

bc

ab

b

b

b

b

ab

c

a

bc

bc

cd

d

c

c

c

c

cd

b

b

a

c

c

c

bcb

ab

d

cd

cd

cd

cd

cd

c

b

**Fig. S2** Continuation

ab

c

c

c

c

d

b

b

a

a

a

a

ab

ab

ab

ab

ab

b

b

b

b

b

a

a

a

d

c

b

a

a

a

a

a

**Fig. S2** Continuation

ab

b

b

b

ab

c

c

c

b

b

a

a

a

a

c

a

a

b

a

a

a

a

bc

b

b

b

ac

ac

bc

bc

b

b

b

b

b

c

bc

d

a

a

a

a

a

a

a

a

**Fig. S2** Continuation

c

a

a

ab

a

a

b

a

a

ac

a

d

bc

bc

c

b

a

a

a

c

b

ab

ab

ab

ab

b

b

b

a

a

a

a

a

a
